# Double-barrel STA-MCA bypass with endovascular parent artery occlusion for complex anterior circulation aneurysms: indications and outcomes

**DOI:** 10.1007/s00701-026-06833-3

**Published:** 2026-03-16

**Authors:** Chingiz Nurimanov, Karashash Menlibayeva, Iroda Mammadinova, Assylbek Kaliyev, Yerbol Makhambetov, Serik Akshulakov

**Affiliations:** 1 Vascular and Functional Neurosurgery Department, National Centre for Neurosurgery, Astana, Kazakhstan; 2 Department of Population Health Sciences, Faculty of Life Sciences and Medicine, King’s College London, London, United Kingdom

**Keywords:** Complex giant aneurysms, Cerebral revascularization, Double-barrel bypass, Parent artery occlusion, Hybrid approach

## Abstract

To assess the long-term clinical outcomes of a hybrid approach combining double-barrel superficial temporal artery to middle cerebral artery (STA-MCA) bypass with endovascular parent artery occlusion in the treatment of giant complex intracranial aneurysms. This retrospective observational study included patients with giant or fusiform intracranial aneurysms who underwent double-barrel STA-MCA bypass followed by endovascular occlusion of the parent artery between January 2019 and January 2025. The primary outcomes were bypass patency, aneurysm exclusion, and ischaemic complications. Secondary outcomes included postoperative neurological status (assessed using the modified Rankin Scale [mRS]), radiological follow-up results, and procedural complications. Follow-up was conducted at 1-, 3-, and 6-month post-procedure and annually thereafter, including clinical assessment and imaging with MRI/MRA or DSA. Seven patients (mean age, 44.7 ± 27.5 years; 57.1% male) were included in the analysis. Most aneurysms were fusiform (85.7%) and located in the MCA (71.4%). All patients underwent double-barrel bypass and endovascular occlusion. Postoperative DSA confirmed aneurysm exclusion and preserved perfusion in all cases. No permanent neurological deficits or bypass failures were observed. One patient developed transient hemiparesis, which was resolved spontaneously without intervention. In one emergency case, a double-barrel bypass was performed following coil prolapse and occlusion of both M2 segments during endovascular embolization; the patient was discharged with mild residual contralateral paresis. At long-term follow-up, all aneurysms remained completely occluded, and bypass patency was maintained in all patients. In appropriately selected cases, a hybrid approach combining double-barrel STA-MCA bypass with endovascular parent artery occlusion offers a safe and effective treatment option for complex intracranial aneurysms. This strategy provides reliable flow restoration and durable aneurysm exclusion, particularly when performed in a hybrid operating setting.

## Introduction

Complex intracranial aneurysms present significant therapeutic challenges, necessitating advanced microsurgical or endovascular strategies and substantial institutional resources. Their management is complicated by a constellation of clinical factors, including lesion size, anatomical location, prior interventions, collateral circulation, presence of intraluminal thrombus, and wall calcification, which collectively increase procedural complexity and increase the suboptimal outcomes risks [3, 13]. Giant aneurysms, a subtype of complex intracranial aneurysms, are frequently associated with mass effect and cerebral ischaemia [11], further complicating the management. Moreover, tortuous vascular anatomy and disrupted haemodynamics are associated with increased rates of postoperative morbidity and mortality [8, 12, 14].

Although flow-diverter stents represent a cornerstone in the treatment of intracranial aneurysms, complex morphological features of giant aneurysms are associated with an increased risk of incomplete occlusion following their use [24]. In such cases, parent artery occlusion combined with cerebral revascularization becomes essential to preserve distal perfusion and mitigate ischaemic risk [1, 25]. A hybrid approach, integrating endovascular treatment with open microsurgical revascularization, offers a viable alternative [2, 21]. The superficial temporal artery to middle cerebral artery (STA-MCA) bypass remains a well-established technique; traditionally performed using a single STA branch to provide low-flow augmentation. However, for complex MCA aneurysms, a double-barrel bypass using both STA branches is often warranted [10]. This approach provides superior flow augmentation and facilitates the revascularization of distinct MCA territories, reduces the risk of ischaemic complications, and improves clinical outcomes, particularly in the management of giant aneurysms in the anterior circulation [10].

Despite its theoretical advantages, the combined use of double-barrel STA-MCA bypass and endovascular parent artery occlusion has been rarely described. In this study, we report our institutional experience employing the hybrid approach, focusing on technical feasibility, clinical outcomes, and its potential role in the evolving management of complex anterior circulation aneurysms. A recent literature update revealed no existing meta-analyses or systematic reviews on this combined technique, highlighting the paucity of available evidence. Over the past decade, substantial progress in microsurgical techniques and endovascular technologies has renewed interest in integrated treatment strategies. We consider that our findings add valuable evidence to this evolving field and may inform future therapeutic frameworks for these complex cerebrovascular conditions.

## Study description

We reviewed all cases between January 2019 and January 2025 in which patients with giant intracranial aneurysms underwent a combined treatment approach involving STA-MCA bypass and endovascular parent artery occlusion.

Seven patients with complex intracranial aneurysms were included in the analysis. The mean age was 44.71 ± 27.51 years, and four (57.14%) were male. Most aneurysms were in the MCA (71.43%) and showed a fusiform morphology (85.71%). Three patients (42.9%) had a history of preoperative stroke or transient ischemic attack (TIA). At long-term follow-up, complete aneurysm occlusion was achieved in all cases. Demographic and clinical characteristics are summarized in Table 1.

**Table 1:** Demographic and clinical characteristics of aneurysm cases

Patients	Age (years)	Gender	Location	Aneurysm characteristics	Aneurysm size	Rupture status	History of stroke	Initial neurologic deficit	Previous surgery	Collaterals	Bypass	Embolization	Complication	Follow-up data
Patient 1	60	male	MCA	fusiform	51.5 × 48.5mm	unruptured	no	vertigo	no	ACom	Double STA-MCA	Coil	no	complete occlusion
Patient 2	74	male	ICA	saccular	47.6 × 46.3mm	unruptured	no	left side 3, 4, 6 nerve palsy and loss of vision	no	no	Double STA-MCA	Coil and Onyx	no	complete occlusion
Patient 3	16	female	MCA	fusiform	28.3 × 25.1 mm	unruptured	TIA	no	no	no	Double STA-MCA	Coil	no	complete occlusion
Patient 4	17	male	MCA	fusiform	34.2 × 32.2mm	ruptured	SAH, right temporal ICH	no	Partial coil embolization	no	Double STA-MCA	Coil	temporary left-sided hemiparesis	complete occlusion
Patient 5	64	female	ICA	fusiform	35.0 × 32.0 mm	ruptured	SAH	no	no	no	Double STA-MCA	Coil	no	complete occlusion
Patient 6	68	female	MCA	saccular	26.5 × 18.5mm	unruptured	no	no	Coil embolization – left MCA territory ischemic stroke	ACom	Emergency Double STA-MCA	Coil	right-side hemiparesis	complete occlusion
Patient 7	14	male	MCA	fusiform	15.2 × 14.5mm	unruptured	no	no	no	no	Double STA-MCA	Coil	no	complete occlusion

### Patient selection

Patients were eligible for inclusion if they met the following criteria: were diagnosed with a giant (≥ 25 mm) or fusiform intracranial aneurysm located in the anterior circulation, confirmed by digital subtraction angiography (DSA) and MRI and underwent a combined treatment approach consisting of a double STA-MCA bypass followed by endovascular parent artery occlusion; were deemed unsuitable for isolated endovascular or microsurgical treatment due to unfavorable morphology, high thrombus burden, or compromised distal flow; and received the hybrid strategy as a planned, staged procedure. Balloon occlusion testing was performed preoperatively in all cases to assess the adequacy of collateral circulation and the safety of parent artery sacrifice. Patients who underwent a hybrid approach involving either a single STA-MCA bypass or a high-flow EC-IC bypass were excluded from the study.

### Surgical and endovascular procedure

All patients first underwent bypass surgery using a double-barrel STA-MCA technique. Both frontal and parietal branches of the STA were anastomosed to separate cortical MCA branches to augment distal flow. Intraoperative indocyanine green (ICG) angiography and micro-Doppler ultrasonography were employed to assess graft patency. Endovascular parent artery occlusion was performed in a second-stage procedure following angiographic confirmation of bypass sufficiency, after the microsurgical intervention. Endovascular occlusion was performed on the same day as the bypass surgery, under general anesthesia, using detachable coils. All patients received antiplatelet therapy with aspirin at a dose of 100 mg per day in the immediate postoperative period, which was continued as long-term treatment.

### Data collection and outcomes

Clinical records, imaging data, operative reports, and postoperative follow-up information were reviewed. The primary outcomes were bypass patency, aneurysm exclusion, and ischaemic complications. Secondary outcomes included postoperative neurological status (assessed using the modified Rankin Scale [mRS]), radiological follow-up results, and procedural complications. Follow-up was conducted at 1-, 3-, and 6-month post-procedure and annually thereafter, including clinical assessment and imaging with MRI/MRA or DSA.

### Indications for double-barrel STA-MCA bypass and endovascular parent artery occlusion

#### Case 1: giant fusiform, partially thrombosed MCA aneurysm

**Fig. 1:**
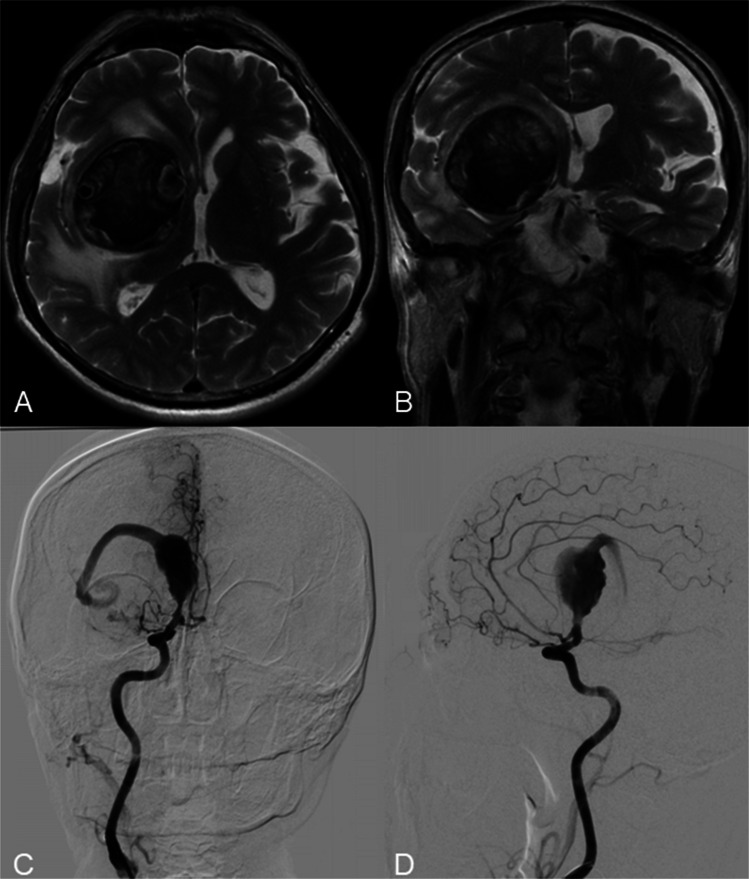
Axial (**A**) and frontal (**B**) T2-weighted brain MRI demonstrating the giant intracranial aneurysm with thrombosis in the lesion and surrounding edema. DSA from the right ICA (**c**, **d**) shows a giant partially thrombosed M1-2 segment fusiform aneurysm of the right middle cerebral artery

A 60-year-old patient presented with persistent headache and vertigo. MRI revealed a giant fusiform aneurysm of the right MCA, measuring 51.5 × 48.5 × 51.1 mm, with intraluminal thrombosis and surrounding oedema involving the frontal, parietal, and temporal lobes (Fig. 1A, B). DSA demonstrated a partially thrombosed fusiform aneurysm extending from the M1 to M2 segments of the right MCA (Fig. 1C, D). Due to the aneurysm’s morphology and size, neither microsurgical clipping nor endovascular embolization was considered feasible. To restore perfusion to the affected MCA territory, a double-barrel extracranial-intracranial bypass was performed using both branches of the superficial temporal artery on the right side. Intraoperative ICG video angiography confirmed immediate graft patency.

**Fig. 2:**
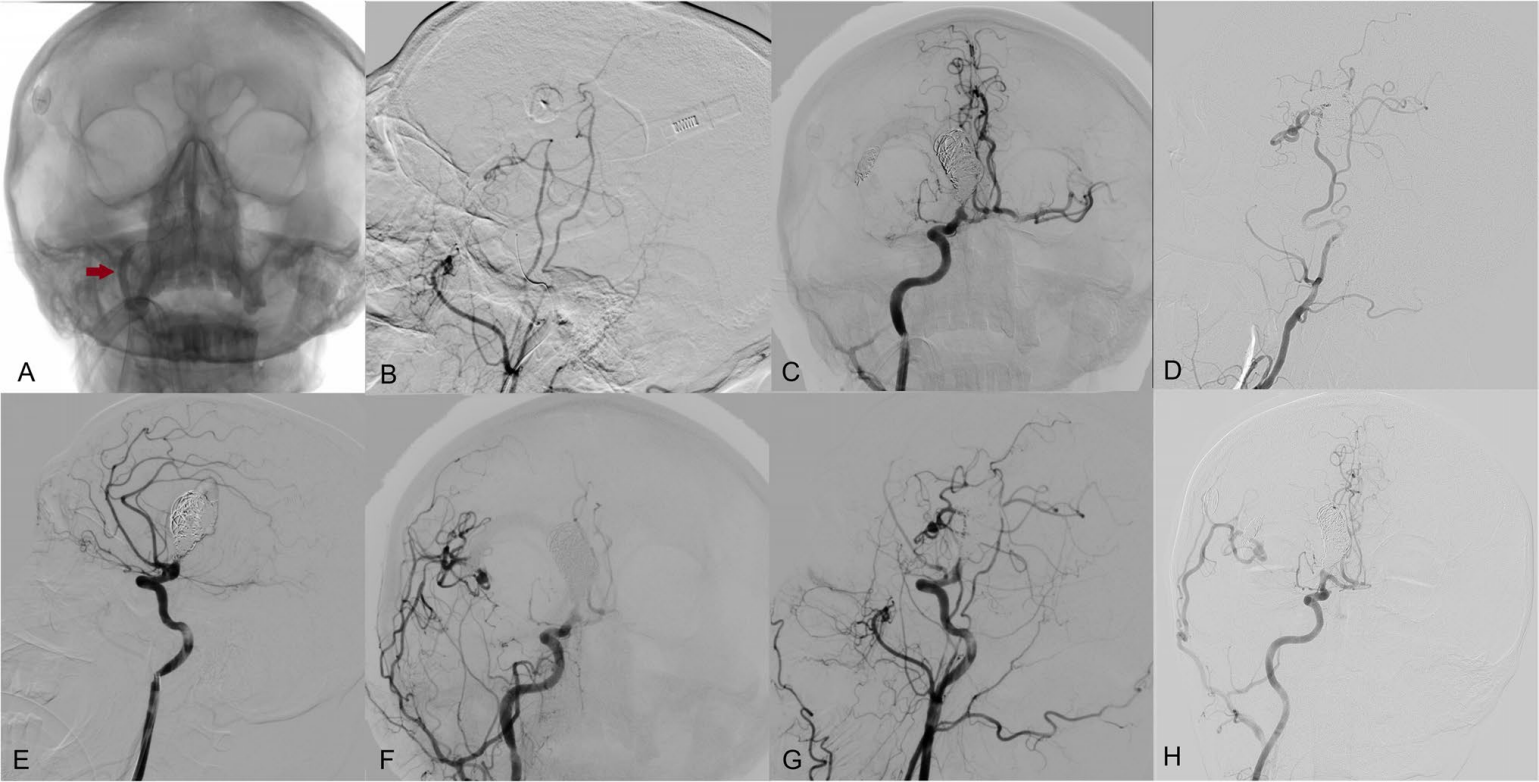
DSA from the right common carotid artery shows balloon occlusion (**A**) of the right ICA (red arrow points to the balloon in ICA) and patency of the double STA-MCA bypass (**B**). DSA from the right ICA (**C**, **E**) shows a coiled proximal and distal part of the M1 fusiform aneurysm of the MCA. DSA from the right common carotid artery shows a coiled aneurysm and patency of the double STA-MCA bypass (**F**, **G**). Two-year follow-up angiogram (**D**, **H**) shows robust bypass flow, complete aneurysm occlusion, and matured frontal STA branch

Postoperative DSA, including balloon occlusion testing of the petrous segment of the right internal carotid artery (ICA), confirmed sufficient flow through the bypass grafts (Fig. 2A, B).

Following confirmation of bypass patency, endovascular coil occlusion of both proximal and distal aspects of the aneurysm was performed (Fig. 2C, E). Final DSA via the right common carotid artery demonstrated complete exclusion of the aneurysm from circulation, with preserved perfusion of the M2 branches via the double-barrel bypass (Fig. 2F, G). A follow-up angiogram performed two years later (Fig. 2D, H) showed good bypass flow and complete aneurysm occlusion, along with maturation of the frontal branch of the STA.

#### Case 2: giant fusiform, partially thrombosed ICA aneurysm with mass effect

**Fig. 3:**
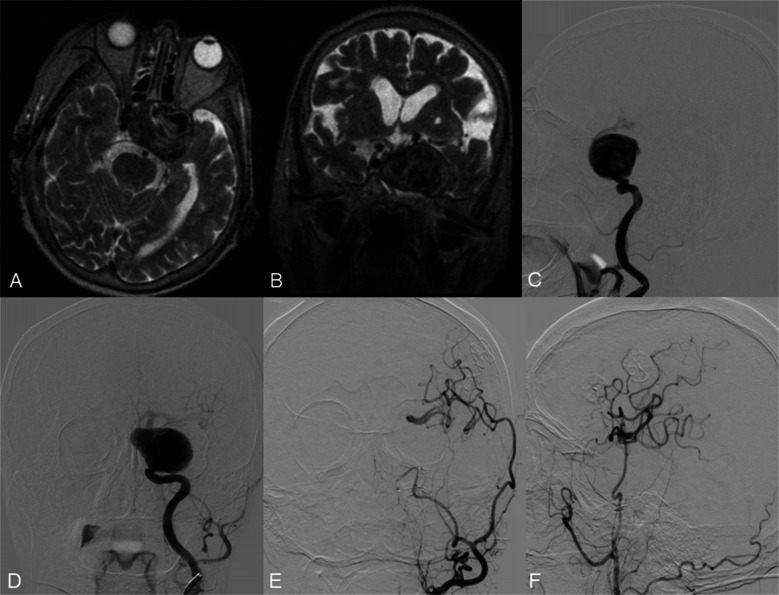
Axial (**A**) and frontal (**B**) T2-weighted brain MRI demonstrating the giant intracranial aneurysm of the left ICA. DSA from the left ICA (**C**, **D**) shows a giant aneurysm of the left ICA. DSA from the left common carotid artery shows patency of the double STA-MCA bypass (**E**, **F**)

A 74-year-old patient presented with a giant unruptured intracranial aneurysm of the left ICA, measuring 47.6 × 46.3 × 36.3 mm. MRI demonstrated partial thrombosis within the lesion (Fig. 3A, B). DSA revealed a complex aneurysm extending from the cavernous to the supraclinoid segment of the left ICA, accompanied by fusiform dilatation of the cavernous segment (Fig. 3C, D).

**Fig. 4:**
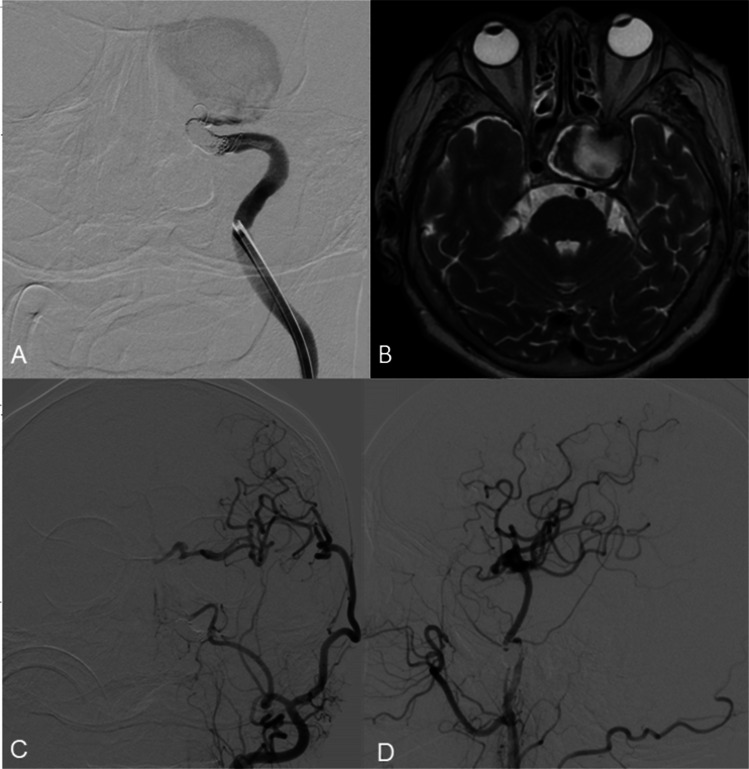
DSA from the left ICA (**A**) shows the coiled proximal and distal parts of the ICA aneurysm. DSA from left CCA (**C**, **D**) shows complete aneurysm occlusion and satisfactory patency of the double STA-MCA bypass after 3 months of follow-up. Three-month MRI (**B**) shows reduced aneurysm size and volume

Given the aneurysm’s size, location, and morphology, a double-barrel STA-MCA bypass was performed on the left side. Intraoperative assessment of graft patency was followed by balloon occlusion testing of the petrous segment of the left ICA, which confirmed adequate collateral flow through the bypass (Fig. 3E, F). Subsequently, endovascular occlusion of the aneurysm was achieved using the detachable coils. (Fig. 4A).

Final DSA from the left common carotid artery demonstrated complete exclusion of the aneurysm and robust perfusion of the M1 segment of the MCA via the double-barrel bypass. At three-month follow-up, DSA confirmed persistent aneurysm occlusion and maintained bypass patency, with satisfactory revascularisation of the MCA territory and partial reconstitution of anterior cerebral artery (ACA) flow (Fig. 4C, D). MRI at three months showed aneurysm shrinkage and a decrease in aneurysmal volume (Fig. 4B).

#### Case 3: giant fusiform MCA aneurysm

**Fig. 5:**
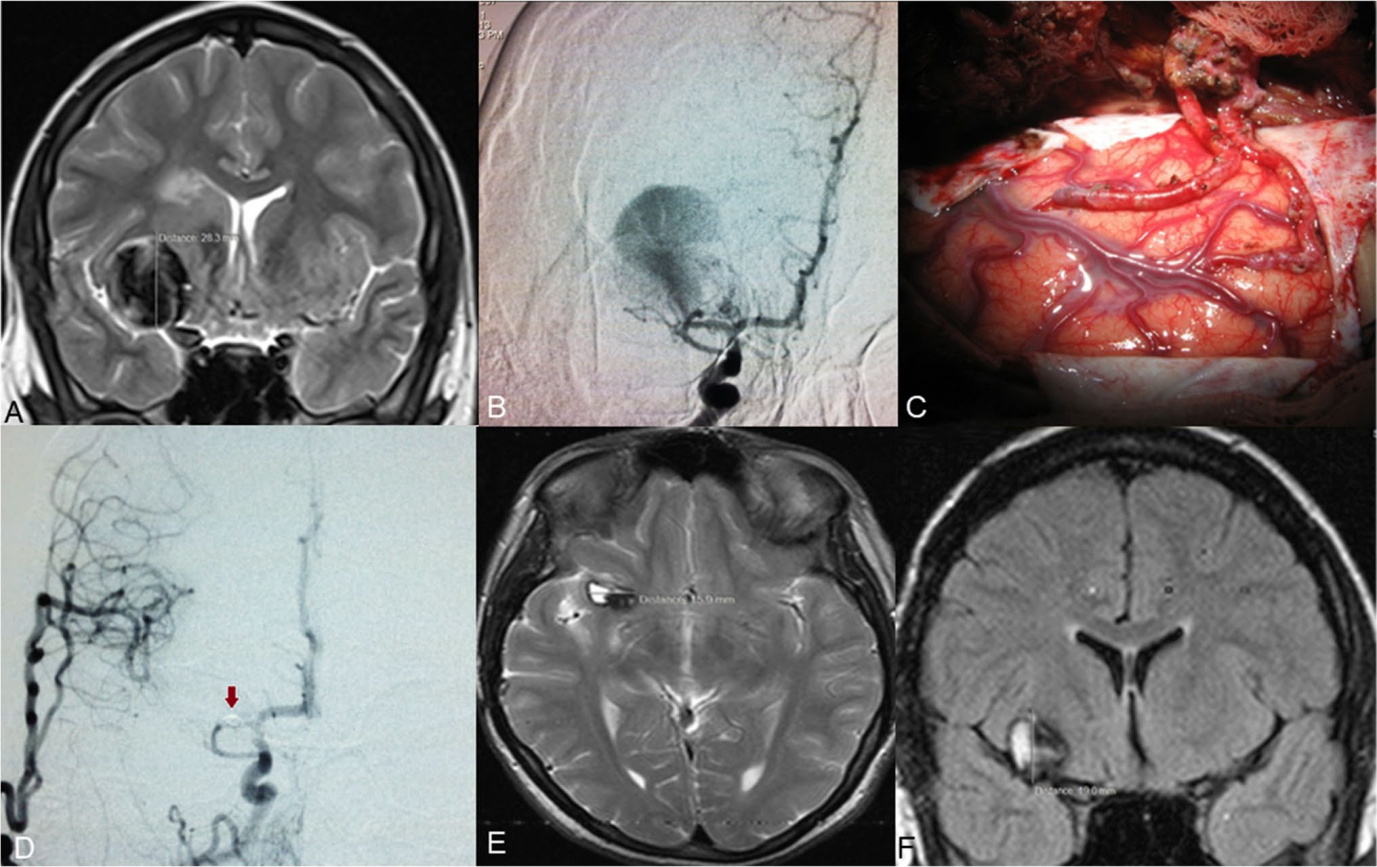
Frontal (**A**) T2-weighted MRI showing the giant intracranial aneurysm of the MCA. DSA from the right ICA (**B**) shows the aneurysm of the M1 segment of the MCA. Intraoperative view showing the STA-MCA (M4 segment) anastomosis (**C**). DSA from right CCA (**D**) demonstrates the total occlusion of the right M1 segment aneurysm and the patency of the double-barrel STA-MCA bypass. Axial (**E**) T2-weighted, and frontal (**F**) brain T1-weighted MRI performed after 9 months, showing the reduction in the size of the aneurysm of the MCA

A 28.3 × 25.1 × 24.7 mm fusiform unruptured aneurysm involving the M1 segment of the right MCA was identified on imaging (Fig. 5A, B). Due to the aneurysm’s morphology and risk of rupture or thromboembolic complications, a double-barrel STA-MCA bypass was performed on the right side to re-establish distal perfusion (Fig. 5C).

Following confirmation of bypass patency, endovascular coil occlusion of the affected M1 segment was undertaken. Post-procedural DSA from the right common carotid artery demonstrated complete exclusion of the aneurysm and satisfactory revascularisation of the M2 territory via the bypass grafts (Fig. 5D).

Neurological examination following surgery revealed no deficits. A follow-up MRI performed three years after treatment confirmed aneurysm shrinkage and the absence of ischemic complications. (Fig. 5E, F).

#### Case 4: giant previously embolized MCA aneurysm

**Fig. 6:**
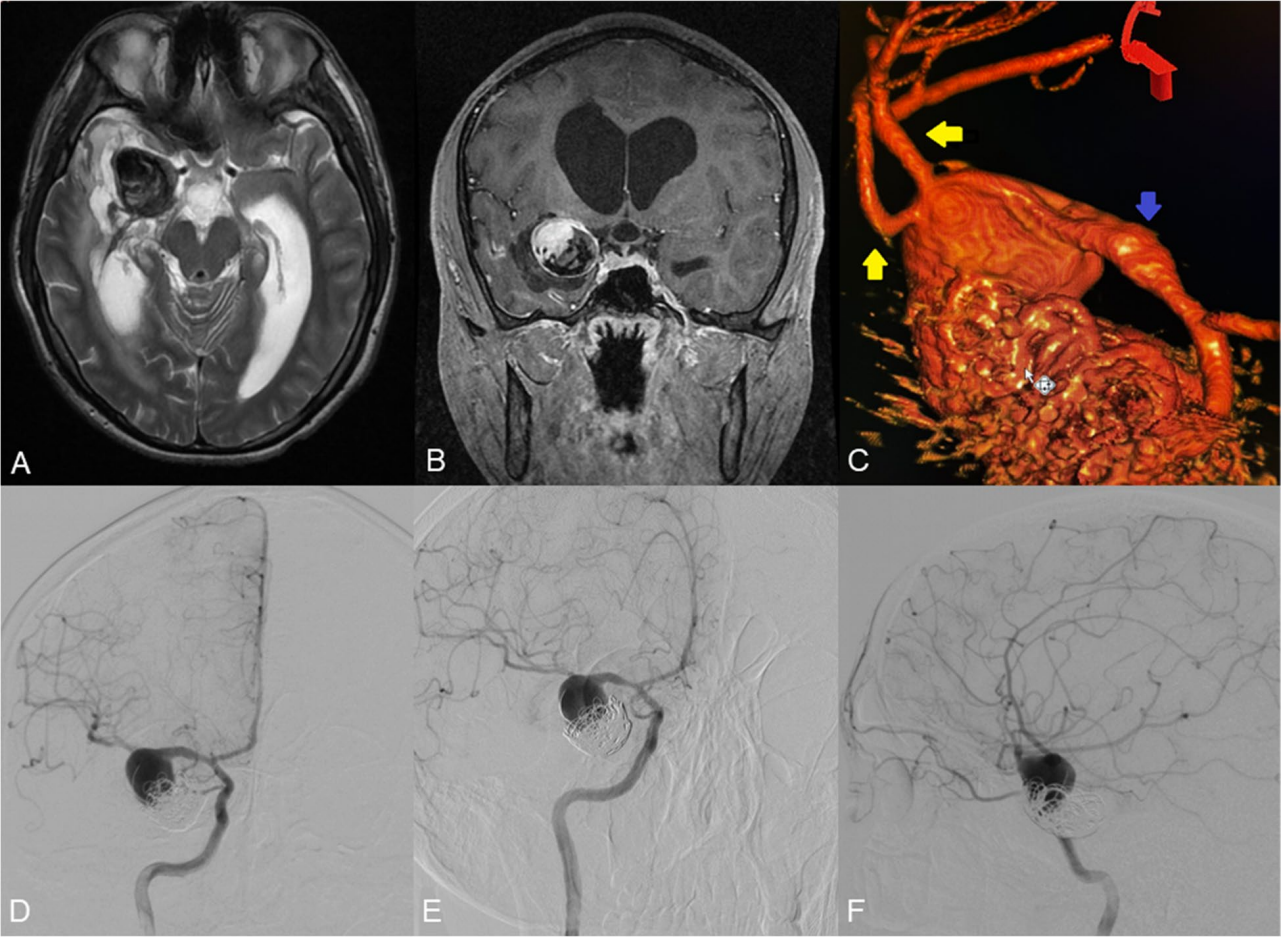
Axial (**A**) T2-weighted and frontal (**B**) T1 contrast-enhanced MRI showing the giant partially thrombosed intracranial aneurysm of the left MCA. **C** 3 d reconstruction of the aneurysm (The blue arrow indicates the M1 segment of MCA, and the yellow arrows indicate the M2 branches of MCA originating from the aneurysm dome). A DSA from the left ICA (**D-F**) shows a giant partially coiled aneurysm of the left MCA

A 34.2 × 32.2 × 29.9 mm giant ruptured aneurysm of the right MCA was identified (Fig. 6A, B). The patient had a history of prior endovascular embolization. Three-dimensional reconstructed DSA confirmed a fusiform morphology, with M2 branches arising directly from the aneurysmal dome (Fig. 6C-F), precluding both further embolization and direct clipping. To restore blood flow, a double-barrel right-sided STA-MCA bypass was performed.

**Fig. 7:**
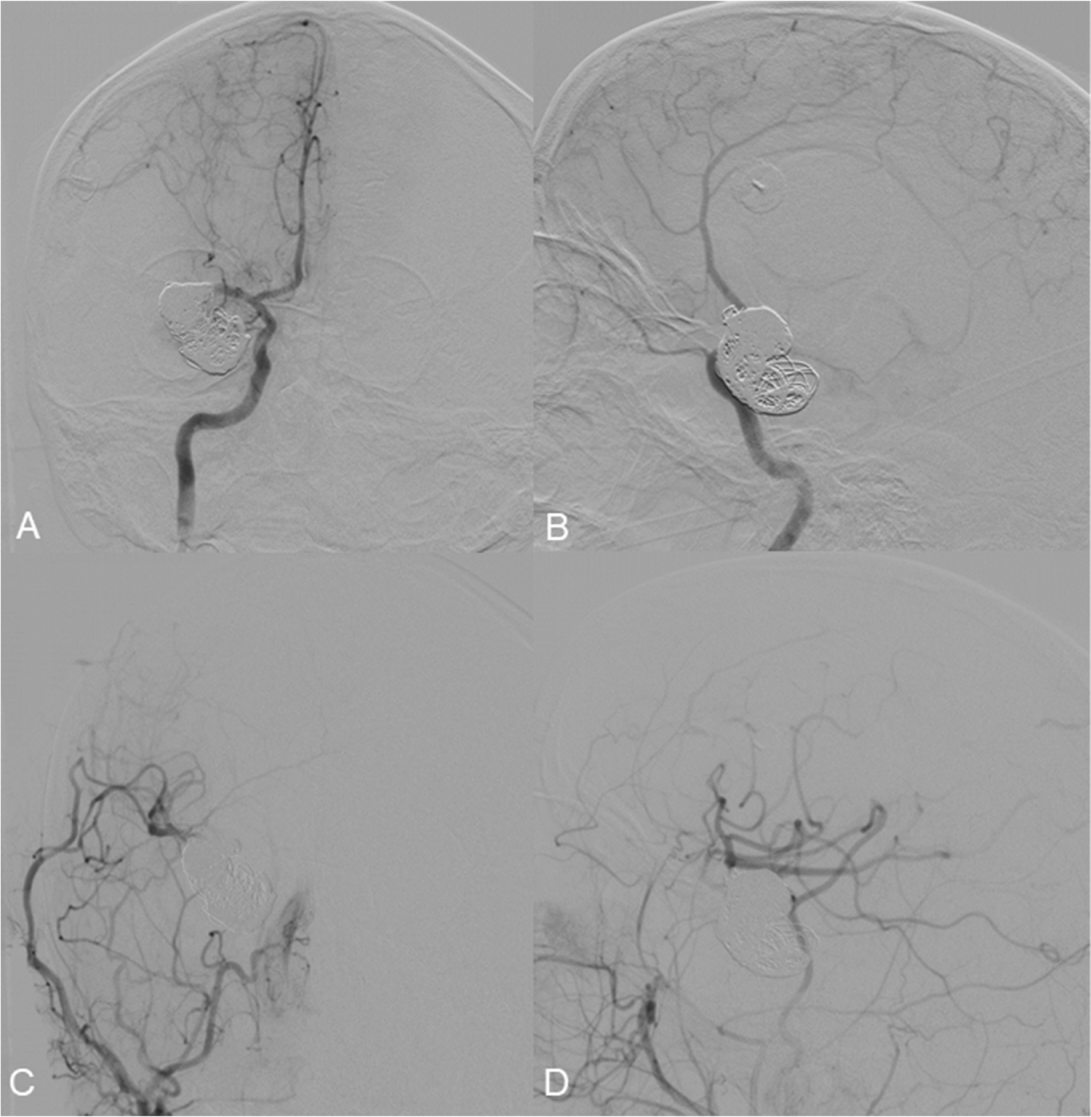
A DSA from left ICA (**A**, **B**) shows a coiled MCA aneurysm and patency of the double STA-MCA bypass (**C**, **D**)

After flow restoration and confirmation of double bypass patency, endovascular coil occlusion of the aneurysm was performed (Fig. 7A, B). Post-procedural DSA from the right common carotid artery confirmed complete aneurysm occlusion and adequate vascularization of the M2 territory via the double-barrel bypass (Fig. 7C, D).

## Discussion

Giant (> 25 mm) and supergiant (> 50 mm) intracranial aneurysms have complex histopathological and haemodynamic features that often necessitate a multimodal treatment strategy, as single-modality approaches are frequently inadequate [6]. Open microsurgical techniques allow for immediate aneurysm exclusion; however, these modalities are often limited in cases of dolichoectatic morphology, aberrant vessel branching, atherosclerotic neck changes, intraluminal thrombus, or prior endovascular coil placement. These factors, all present in our case series, are known to increase the procedural risks associated with open microsurgical interventions [16].

Traditional endovascular interventions, including coiling and stent-assisted coiling, also present limitations. Large coil masses can occlude parent vessels, and recurrence rates remain high, particularly in large and giant aneurysms [7]. Flow diversion, while increasingly used, depends on delayed aneurysm thrombosis and vascular remodeling, which may paradoxically result in continued aneurysm growth and worsening mass effect [4, 9]. Moreover, fusiform MCA aneurysms present a particular challenge for flow diversion, due to the absence of a normal parent artery wall for device anchoring and the proximity of vital perforating vessels [15].

In fusiform MCA aneurysms, stent-assisted coiling and flow diversion techniques have limited effectiveness because they lack a vessel wall for support. Moreover, the requirement for dual antiplatelet medication in patients receiving flow diversion limits its use in acute cases, complicating the management of these complex aneurysms [20]. Additionally, the placement of stents or flow diverters carries a high risk of occluding lenticulostriate arteries and ischemic stroke. Since the origins of the M2 branches are involved in the aneurysm, the stent placement often needs to extend into one of the M2 branches, compromising the other branches and potentially reducing critical blood flow. These risks were evident in our cases 1, 3, and 4.

For such complex aneurysms, reconstructive and deconstructive approaches, including trapping or proximal/distal parent artery occlusion combined with cerebral revascularization, have emerged as effective alternatives. These methods are particularly useful in patients with limited collateral circulation [20, 25]. Trapping and bypass revascularization are effective, as proximal vessel occlusion decreases blood flow through the aneurysm, facilitating thrombosis. Simultaneously, the bypass restores distal perfusion and enables retrograde flow to preserve perforator circulation [25].

Several factors, including vessel caliber, site of occlusion, lenticulostriate artery involvement, and rupture status, determine bypass selection. Tayebi et al. proposed an algorithm for choosing between extracranial-to-intracranial (EC-IC) and intracranial-to-intracranial (IC-IC) bypass techniques [19]. Complete trapping is contraindicated in aneurysms involving the lenticulostriate arteries, as it poses a risk of ischemic complications in the perforating vessels, particularly at prebifurcation or bifurcation sites. In such cases, partial trapping combined with proximal and distal parent artery occlusion may offer a viable therapeutic option. Proximal occlusion reduces aneurysmal flow, facilitating thrombosis, while the bypass restores distal perfusion and supports retrograde flow to preserve perforator circulation. Endovascular parent artery occlusion is particularly advantageous in cases with a high risk of ischemic events in perforating arteries. It allows for real-time assessment of bypass patency and cerebral perfusion, as well as balloon occlusion testing before parent artery sacrifice. While it may not immediately reduce aneurysm-related mass effect, its safety profile makes it the preferred approach in complex aneurysms.

Accurate determination of flow demand and donor vessel suitability is essential in revascularization planning. Donor graft options vary from the superficial temporal artery or occipital artery to interposition grafts employing conduits such as the radial artery or saphenous vein. The selection of a grafting method is often determined through speculative means, relying on expected territorial flows or preoperative assessments like balloon occlusion testing to infer the anticipated flow that requires replacement [2, 17].

The STA-MCA bypass, first described by Yaşargil and Donaghy in the 1960 s, remains widely used for the management of large aneurysms, moyamoya disease, and other cerebrovascular conditions [26]. As a low-flow bypass, it is typically sufficient for territories with preserved collateral supply. However, when larger flow volumes are required, particularly following parent artery occlusion, high-flow grafts such as the radial artery or saphenous vein are preferred. These provide 40–140 mL/min of flow, but are associated with greater surgical morbidity and limitations, such as unsuitability of the radial artery in patients with a positive Allen test [17, 22].

The double-barrel STA-MCA bypass involves both STA branches, significantly increasing bypass capacity compared to single-barrel techniques (mean bypass outflow: 69 mL/min vs 39 mL/min) [10]. This configuration permits simultaneous revascularization of both frontal and temporal MCA territories, making it a robust option for managing anterior circulation aneurysms requiring extensive flow restoration [5, 10, 23]. In our series, no patients experienced skin necrosis, a known risk with this approach, particularly during secondary procedures [18].

Only one patient (case 4) experienced transient postoperative hemiparesis, which resolved spontaneously. This finding aligns with previously published data demonstrating the favorable risk profile of combined microsurgical and endovascular strategies [27]. In our experience, the main indications for the hybrid approach included giant size, fusiform morphology, direct involvement of M2 branches, or previous coil embolisation in case 4, factors that significantly increase the risk profile of standalone microsurgical or endovascular therapy.

Of particular note, Cases 2 and 3 demonstrated significant aneurysm shrinkage, and in Case 2, resolution of cranial nerve deficits following treatment, highlighting the potential clinical benefits of early and effective relief of mass effect. Case 6 demonstrated the utility of emergency double-barrel bypass, performed within five hours after endovascular coil prolapse led to occlusion of both M2 segments. Although the patient was discharged with mild residual paresis, the bypass successfully preserved critical cerebral perfusion, underscoring its value as a salvage strategy in managing iatrogenic complications.

## Conclusion

The combined use of double-barrel STA-MCA bypass and endovascular parent artery occlusion remains a rarely reported strategy for the treatment of complex intracranial aneurysms. However, in carefully selected patients, this hybrid approach, particularly when performed in a dedicated hybrid operating suite, can achieve effective flow restoration and favorable clinical outcomes. Further studies and accumulated clinical experience are needed to define its indications better and optimize its role in the multidisciplinary management of complex cerebrovascular lesions.

## Data Availability

No datasets were generated or analysed during the current study.
